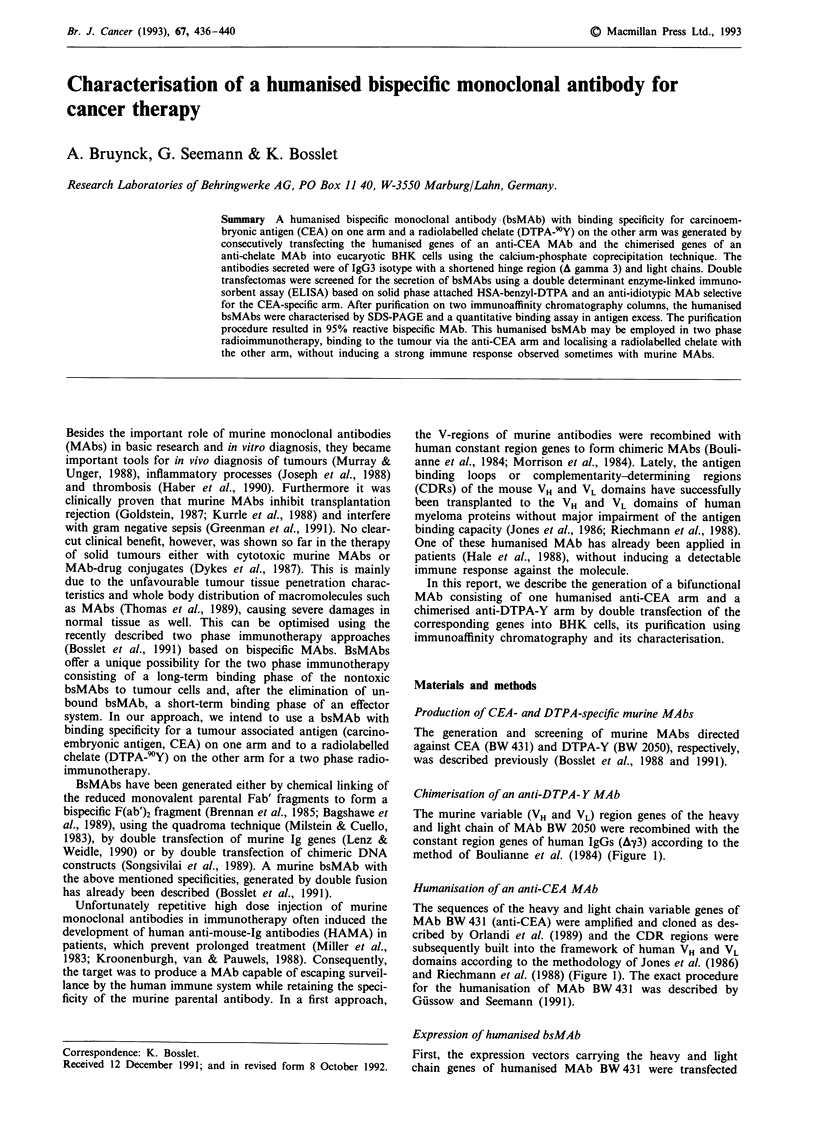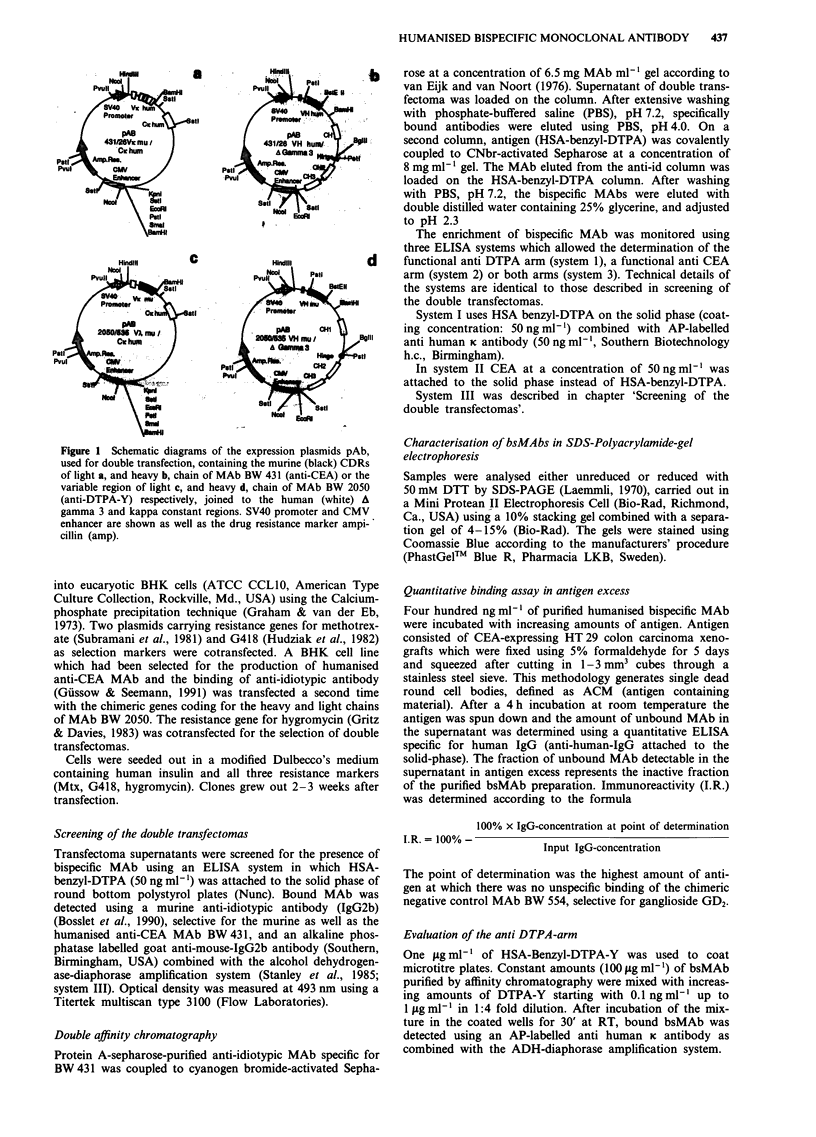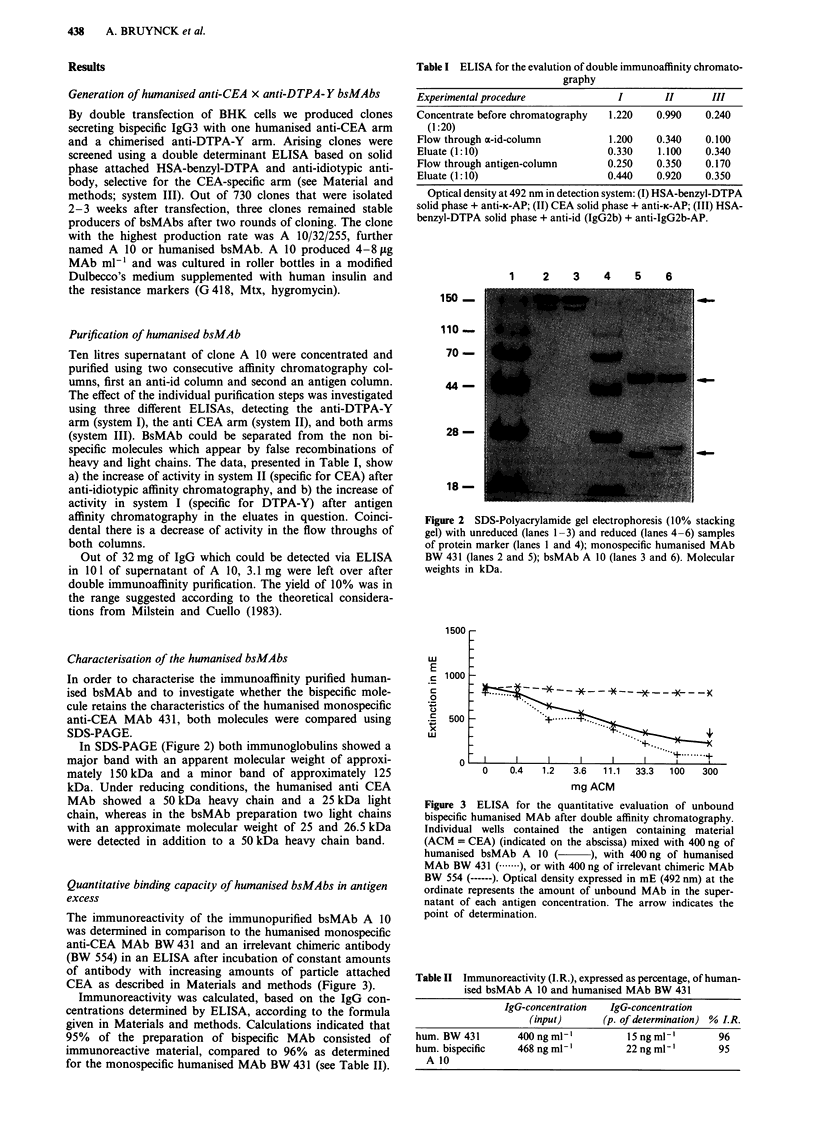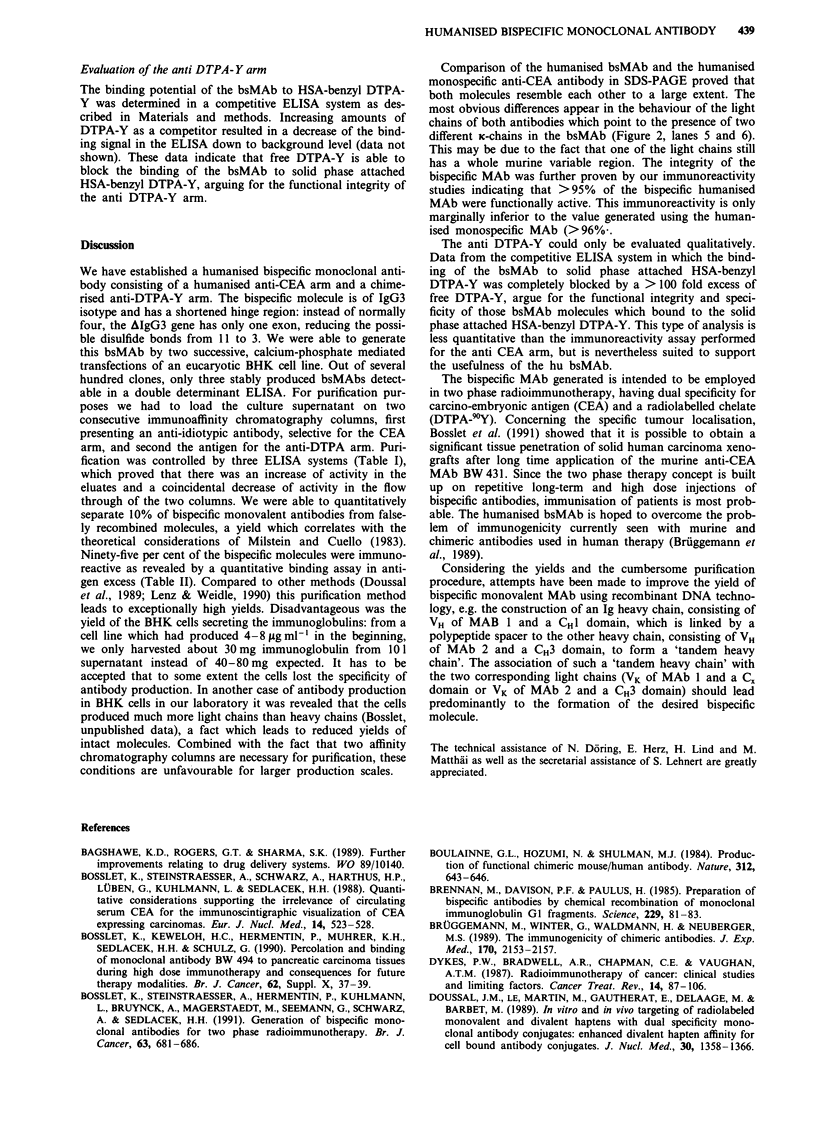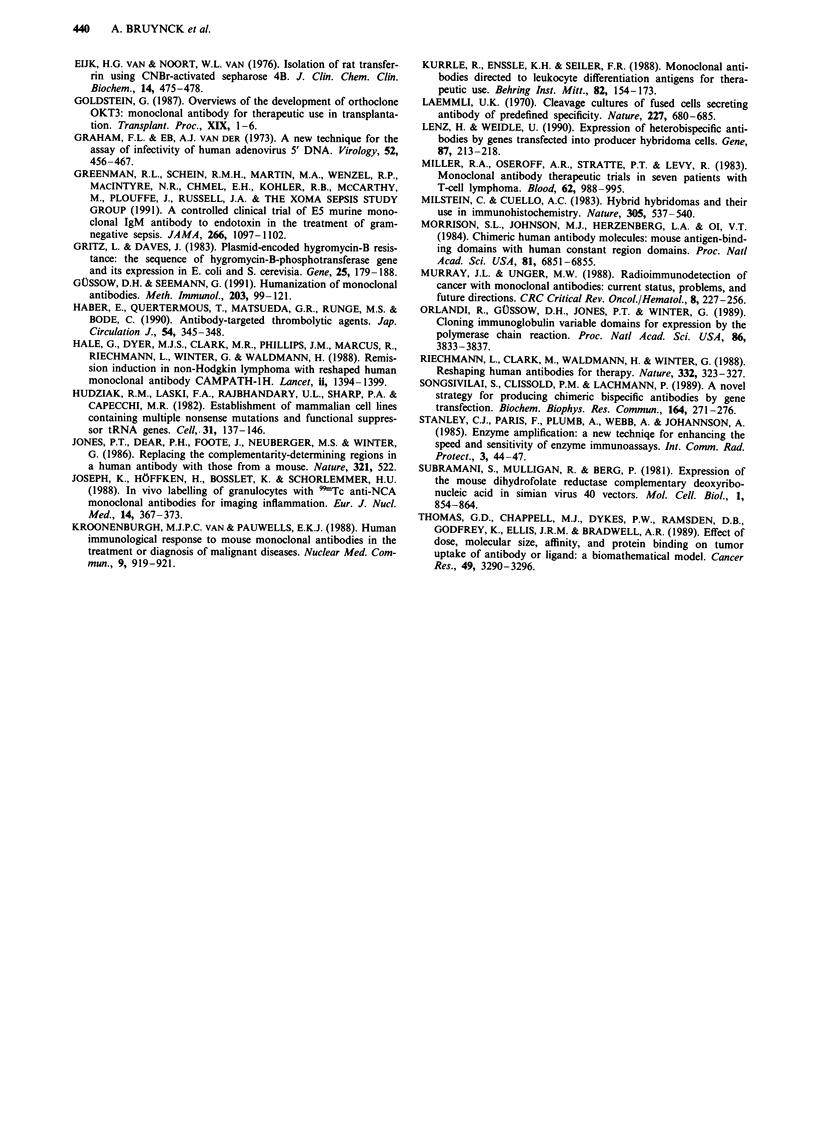# Characterisation of a humanised bispecific monoclonal antibody for cancer therapy.

**DOI:** 10.1038/bjc.1993.84

**Published:** 1993-03

**Authors:** A. Bruynck, G. Seemann, K. Bosslet

**Affiliations:** Research Laboratories of Behringwerke AG, Marburg/Lahn, Germany.

## Abstract

**Images:**


Br. J. Cancer (1993), 67, 436-440 © Macmillan Press Ltd., 1993

### Characterisation of a humanised bispecific monoclonal antibody for cancer therapy

A. Bruynck, G. Seemann & K. Bosslet

*Research Laboratories of Behringwerke AG, PO Box 11 40, W-3550 Marburg/Lahn, Germany.*

**Summary** A humanised bispecific monoclonal antibody (bsMAb) with binding specificity for carcinoembryonic antigen (CEA) on one arm and a radiolabelled chelate (DTPA-$^{90}$Y) on the other arm was generated by consecutively transfecting the humanised genes of an anti-CEA MAb and the chimerised genes of an anti-chelate MAb into eucaryotic BHK cells using the calcium-phosphate coprecipitation technique. The antibodies secreted were of IgG3 isotype with a shortened hinge region (Δ gamma 3) and light chains. Double transfectomas were screened for the secretion of bsMAbs using a double determinant enzyme-linked immunosorbent assay (ELISA) based on solid phase attached HSA-benzyl-DTPA and an anti-idiotypic MAb selective for the CEA-specific arm. After purification on two immunoaffinity chromatography columns, the humanised bsMAbs were characterised by SDS-PAGE and a quantitative binding assay in antigen excess. The purification procedure resulted in 95% reactive bispecific MAb. This humanised bsMAb may be employed in two phase radioimmunotherapy, binding to the tumour via the anti-CEA arm and localising a radiolabelled chelate with the other arm, without inducing a strong immune response observed sometimes with murine MAbs.

Besides the important role of murine monoclonal antibodies (MAbs) in basic research and *in vitro* diagnosis, they became important tools for *in vivo* diagnosis of tumours (Murray & Unger, 1988), inflammatory processes (Joseph *et al.*, 1988) and thrombosis (Haber *et al.*, 1990). Furthermore it was clinically proven that murine MAbs inhibit transplantation rejection (Goldstein, 1987; Kurrle *et al.*, 1988) and interfere with gram negative sepsis (Greenman *et al.*, 1991). No clear-cut clinical benefit, however, was shown so far in the therapy of solid tumours either with cytotoxic murine MAbs or MAb-drug conjugates (Dykes *et al.*, 1987). This is mainly due to the unfavourable tumour tissue penetration characteristics and whole body distribution of macromolecules such as MAbs (Thomas *et al.*, 1989), causing severe damages in normal tissue as well. This can be optimised using the recently described two phase immunotherapy approaches (Bosslet *et al.*, 1991) based on bispecific MAbs. BsMAbs offer a unique possibility for the two phase immunotherapy consisting of a long-term binding phase of the nontoxic bsMAbs to tumour cells and, after the elimination of unbound bsMAb, a short-term binding phase of an effector system. In our approach, we intend to use a bsMAb with binding specificity for a tumour associated antigen (carcinoembryonic antigen, CEA) on one arm and to a radiolabelled chelate (DTPA-$^{90}$Y) on the other arm for a two phase radioimmunotherapy.

BsMAbs have been generated either by chemical linking of the reduced monovalent parental Fab' fragments to form a bispecific F(ab')$_2$ fragment (Brennan *et al.*, 1985; Bagshawe *et al.*, 1989), using the quadroma technique (Milstein & Cuello, 1983), by double transfection of murine Ig genes (Lenz & Weidle, 1990) or by double transfection of chimeric DNA constructs (Songsivilai *et al.*, 1989). A murine bsMAb with the above mentioned specificities, generated by double fusion has already been described (Bosslet *et al.*, 1991).

Unfortunately repetitive high dose injection of murine monoclonal antibodies in immunotherapy often induced the development of human anti-mouse-Ig antibodies (HAMA) in patients, which prevent prolonged treatment (Miller *et al.*, 1983; Kroonenburgh, van & Pauwels, 1988). Consequently, the target was to produce a MAb capable of escaping surveillance by the human immune system while retaining the specificity of the murine parental antibody. In a first approach, the V-regions of murine antibodies were recombined with human constant region genes to form chimeric MAbs (Boulianne *et al.*, 1984; Morrison *et al.*, 1984). Lately, the antigen binding loops or complementarity-determining regions (CDRs) of the mouse $V_H$ and $V_L$ domains have successfully been transplanted to the $V_H$ and $V_L$ domains of human myeloma proteins without major impairment of the antigen binding capacity (Jones *et al.*, 1986; Riechmann *et al.*, 1988). One of these humanised MAb has already been applied in patients (Hale *et al.*, 1988), without inducing a detectable immune response against the molecule.

In this report, we describe the generation of a bifunctional MAb consisting of one humanised anti-CEA arm and a chimerised anti-DTPA-Y arm by double transfection of the corresponding genes into BHK cells, its purification using immunoaffinity chromatography and its characterisation.

### Materials and methods

### *Production of CEA- and DTPA-specific murine MAbs*

The generation and screening of murine MAbs directed against CEA (BW 431) and DTPA-Y (BW 2050), respectively, was described previously (Bosslet *et al.*, 1988 and 1991).

### *Chimerisation of an anti-DTPA-Y MAb*

The murine variable ($V_H$ and $V_L$) region genes of the heavy and light chain of MAb BW 2050 were recombined with the constant region genes of human IgGs (Δγ3) according to the method of Boulianne *et al.* (1984) (Figure 1).

### *Humanisation of an anti-CEA MAb*

The sequences of the heavy and light chain variable genes of MAb BW 431 (anti-CEA) were amplified and cloned as described by Orlandi *et al.* (1989) and the CDR regions were subsequently built into the framework of human $V_H$ and $V_L$ domains according to the methodology of Jones *et al.* (1986) and Riechmann *et al.* (1988) (Figure 1). The exact procedure for the humanisation of MAb BW 431 was described by Güssow and Seemann (1991).

### *Expression of humanised bsMAb*

First, the expression vectors carrying the heavy and light chain genes of humanised MAb BW 431 were transfected

Correspondence: K. Bosslet.
Received 12 December 1991; and in revised form 8 October 1992.

**Figure 1** Schematic diagrams of the expression plasmids pAb, used for double transfection, containing the murine (black) CDRs of light **a**, and heavy **b**, chain of MAb BW 431 (anti-CEA) or the variable region of light **c**, and heavy **d**, chain of MAb BW 2050 (anti-DTPA-Y) respectively, joined to the human (white) **Δ** gamma 3 and kappa constant regions. SV40 promoter and CMV enhancer are shown as well as the drug resistance marker ampicillin (amp).

into eucaryotic BHK cells (ATCC CCL10, American Type Culture Collection, Rockville, Md., USA) using the Calcium-phosphate precipitation technique (Graham & van der Eb, 1973). Two plasmids carrying resistance genes for methotrexate (Subramani *et al.*, 1981) and G418 (Hudziak *et al.*, 1982) as selection markers were cotransfected. A BHK cell line which had been selected for the production of humanised anti-CEA MAb and the binding of anti-idiotypic antibody (Güssow & Seemann, 1991) was transfected a second time with the chimeric genes coding for the heavy and light chains of MAb BW 2050. The resistance gene for hygromycin (Gritz & Davies, 1983) was cotransfected for the selection of double transfectomas.

Cells were seeded out in a modified Dulbecco's medium containing human insulin and all three resistance markers (Mtx, G418, hygromycin). Clones grew out 2–3 weeks after transfection.

*Screening of the double transfectomas*

Transfectoma supernatants were screened for the presence of bispecific MAb using an ELISA system in which HSA-benzyl-DTPA (50 ng $ml^{-1}$) was attached to the solid phase of round bottom polystyrol plates (Nunc). Bound MAb was detected using a murine anti-idiotypic antibody (IgG2b) (Bosslet *et al.*, 1990), selective for the murine as well as the humanised anti-CEA MAb BW 431, and an alkaline phosphatase labelled goat anti-mouse-IgG2b antibody (Southern, Birmingham, USA) combined with the alcohol dehydrogenase-diaphorase amplification system (Stanley *et al.*, 1985; system III). Optical density was measured at 493 nm using a Titertek multiscan type 3100 (Flow Laboratories).

*Double affinity chromatography*

Protein A-sepharose-purified anti-idiotypic MAb specific for BW 431 was coupled to cyanogen bromide-activated Sepharose at a concentration of 6.5 mg MAb $ml^{-1}$ gel according to van Eijk and van Noort (1976). Supernatant of double transfectoma was loaded on the column. After extensive washing with phosphate-buffered saline (PBS), pH 7.2, specifically bound antibodies were eluted using PBS, pH 4.0. On a second column, antigen (HSA-benzyl-DTPA) was covalently coupled to CNbr-activated Sepharose at a concentration of 8 mg $ml^{-1}$ gel. The MAb eluted from the anti-id column was loaded on the HSA-benzyl-DTPA column. After washing with PBS, pH 7.2, the bispecific MAbs were eluted with double distilled water containing 25% glycerine, and adjusted to pH 2.3

The enrichment of bispecific MAb was monitored using three ELISA systems which allowed the determination of the functional anti DTPA arm (system 1), a functional anti CEA arm (system 2) or both arms (system 3). Technical details of the systems are identical to those described in screening of the double transfectomas.

System I uses HSA benzyl-DTPA on the solid phase (coating concentration: 50 ng $ml^{-1}$) combined with AP-labelled anti human κ antibody (50 ng $ml^{-1}$, Southern Biotechnology h.c., Birmingham).

In system II CEA at a concentration of 50 ng $ml^{-1}$ was attached to the solid phase instead of HSA-benzyl-DTPA.

System III was described in chapter 'Screening of the double transfectomas'.

*Characterisation of bsMAbs in SDS-Polyacrylamide-gel electrophoresis*

Samples were analysed either unreduced or reduced with 50 mM DTT by SDS-PAGE (Laemmli, 1970), carried out in a Mini Protean II Electrophoresis Cell (Bio-Rad, Richmond, Ca., USA) using a 10% stacking gel combined with a separation gel of 4–15% (Bio-Rad). The gels were stained using Coomassie Blue according to the manufacturers' procedure (PhastGel™ Blue R, Pharmacia LKB, Sweden).

*Quantitative binding assay in antigen excess*

Four hundred ng $ml^{-1}$ of purified humanised bispecific MAb were incubated with increasing amounts of antigen. Antigen consisted of CEA-expressing HT 29 colon carcinoma xenografts which were fixed using 5% formaldehyde for 5 days and squeezed after cutting in 1–3 $mm^3$ cubes through a stainless steel sieve. This methodology generates single dead round cell bodies, defined as ACM (antigen containing material). After a 4 h incubation at room temperature the antigen was spun down and the amount of unbound MAb in the supernatant was determined using a quantitative ELISA specific for human IgG (anti-human-IgG attached to the solid-phase). The fraction of unbound MAb detectable in the supernatant in antigen excess represents the inactive fraction of the purified bsMAb preparation. Immunoreactivity (I.R.) was determined according to the formula

$$\text{I.R.} = 100\% - \frac{100\% \times \text{IgG-concentration at point of determination}}{\text{Input IgG-concentration}}$$

The point of determination was the highest amount of antigen at which there was no unspecific binding of the chimeric negative control MAb BW 554, selective for ganglioside $GD_2$.

*Evaluation of the anti DTPA-arm*

One μg $ml^{-1}$ of HSA-Benzyl-DTPA-Y was used to coat microtitre plates. Constant amounts (100 μg $ml^{-1}$) of bsMAb purified by affinity chromatography were mixed with increasing amounts of DTPA-Y starting with 0.1 ng $ml^{-1}$ up to 1 μg $ml^{-1}$ in 1:4 fold dilution. After incubation of the mixture in the coated wells for 30′ at RT, bound bsMAb was detected using an AP-labelled anti human κ antibody as combined with the ADH-diaphorase amplification system.

### Results

### *Generation of humanised anti-CEA × anti-DTPA-Y bsMAbs*

By double transfection of BHK cells we produced clones secreting bispecific IgG3 with one humanised anti-CEA arm and a chimerised anti-DTPA-Y arm. Arising clones were screened using a double determinant ELISA based on solid phase attached HSA-benzyl-DTPA and anti-idiotypic antibody, selective for the CEA-specific arm (see Material and methods; system III). Out of 730 clones that were isolated 2–3 weeks after transfection, three clones remained stable producers of bsMAbs after two rounds of cloning. The clone with the highest production rate was A 10/32/255, further named A 10 or humanised bsMAb. A 10 produced 4–8 μg MAb $ml^{-1}$ and was cultured in roller bottles in a modified Dulbecco's medium supplemented with human insulin and the resistance markers (G 418, Mtx, hygromycin).

### *Purification of humanised bsMAb*

Ten litres supernatant of clone A 10 were concentrated and purified using two consecutive affinity chromatography columns, first an anti-id column and second an antigen column. The effect of the individual purification steps was investigated using three different ELISAs, detecting the anti-DTPA-Y arm (system I), the anti CEA arm (system II), and both arms (system III). BsMAb could be separated from the non bispecific molecules which appear by false recombinations of heavy and light chains. The data, presented in Table I, show a) the increase of activity in system II (specific for CEA) after anti-idiotypic affinity chromatography, and b) the increase of activity in system I (specific for DTPA-Y) after antigen affinity chromatography in the eluates in question. Coincidental there is a decrease of activity in the flow throughs of both columns.

Out of 32 mg of IgG which could be detected via ELISA in 10 l of supernatant of A 10, 3.1 mg were left over after double immunoaffinity purification. The yield of 10% was in the range suggested according to the theoretical considerations from Milstein and Cuello (1983).

### *Characterisation of the humanised bsMAbs*

In order to characterise the immunoaffinity purified humanised bsMAb and to investigate whether the bispecific molecule retains the characteristics of the humanised monospecific anti-CEA MAb 431, both molecules were compared using SDS-PAGE.

In SDS-PAGE (Figure 2) both immunoglobulins showed a major band with an apparent molecular weight of approximately 150 kDa and a minor band of approximately 125 kDa. Under reducing conditions, the humanised anti CEA MAb showed a 50 kDa heavy chain and a 25 kDa light chain, whereas in the bsMAb preparation two light chains with an approximate molecular weight of 25 and 26.5 kDa were detected in addition to a 50 kDa heavy chain band.

### *Quantitative binding capacity of humanised bsMAbs in antigen excess*

The immunoreactivity of the immunopurified bsMAb A 10 was determined in comparison to the humanised monospecific anti-CEA MAb BW 431 and an irrelevant chimeric antibody (BW 554) in an ELISA after incubation of constant amounts of antibody with increasing amounts of particle attached CEA as described in Materials and methods (Figure 3).

Immunoreactivity was calculated, based on the IgG concentrations determined by ELISA, according to the formula given in Materials and methods. Calculations indicated that 95% of the preparation of bispecific MAb consisted of immunoreactive material, compared to 96% as determined for the monospecific humanised MAb BW 431 (see Table II).

**Table I** ELISA for the evalution of double immunoaffinity chromatography

| *Experimental procedure* | *I* | *II* | *III* |
|---|---|---|---|
| Concentrate before chromatography (1:20) | 1.220 | 0.990 | 0.240 |
| Flow through α-id-column | 1.200 | 0.340 | 0.100 |
| Eluate (1:10) | 0.330 | 1.100 | 0.340 |
| Flow through antigen-column | 0.250 | 0.350 | 0.170 |
| Eluate (1:10) | 0.440 | 0.920 | 0.350 |

Optical density at 492 nm in detection system: (I) HSA-benzyl-DTPA solid phase + anti-κ-AP; (II) CEA solid phase + anti-κ-AP; (III) HSA-benzyl-DTPA solid phase + anti-id (IgG2b) + anti-IgG2b-AP.

**Figure 2** SDS-Polyacrylamide gel electrophoresis (10% stacking gel) with unreduced (lanes 1–3) and reduced (lanes 4–6) samples of protein marker (lanes 1 and 4); monospecific humanised MAb BW 431 (lanes 2 and 5); bsMAb A 10 (lanes 3 and 6). Molecular weights in kDa.

**Figure 3** ELISA for the quantitative evaluation of unbound bispecific humanised MAb after double affinity chromatography. Individual wells contained the antigen containing material (ACM = CEA) (indicated on the abscissa) mixed with 400 ng of humanised bsMAb A 10 (———), with 400 ng of humanised MAb BW 431 (······), or with 400 ng of irrelevant chimeric MAb BW 554 (------). Optical density expressed in mE (492 nm) at the ordinate represents the amount of unbound MAb in the supernatant of each antigen concentration. The arrow indicates the point of determination.

**Table II** Immunoreactivity (I.R.), expressed as percentage, of humanised bsMAb A 10 and humanised MAb BW 431

| | *IgG-concentration (input)* | *IgG-concentration (p. of determination)* | *% I.R.* |
|---|---|---|---|
| hum. BW 431 | 400 ng $ml^{-1}$ | 15 ng $ml^{-1}$ | 96 |
| hum. bispecific A 10 | 468 ng $ml^{-1}$ | 22 ng $ml^{-1}$ | 95 |

*Evaluation of the anti DTPA-Y arm*

The binding potential of the bsMAb to HSA-benzyl DTPA-Y was determined in a competitive ELISA system as described in Materials and methods. Increasing amounts of DTPA-Y as a competitor resulted in a decrease of the binding signal in the ELISA down to background level (data not shown). These data indicate that free DTPA-Y is able to block the binding of the bsMAb to solid phase attached HSA-benzyl DTPA-Y, arguing for the functional integrity of the anti DTPA-Y arm.

### Discussion

We have established a humanised bispecific monoclonal antibody consisting of a humanised anti-CEA arm and a chimerised anti-DTPA-Y arm. The bispecific molecule is of IgG3 isotype and has a shortened hinge region: instead of normally four, the ΔIgG3 gene has only one exon, reducing the possible disulfide bonds from 11 to 3. We were able to generate this bsMAb by two successive, calcium-phosphate mediated transfections of an eucaryotic BHK cell line. Out of several hundred clones, only three stably produced bsMAbs detectable in a double determinant ELISA. For purification purposes we had to load the culture supernatant on two consecutive immunoaffinity chromatography columns, first presenting an anti-idiotypic antibody, selective for the CEA arm, and second the antigen for the anti-DTPA arm. Purification was controlled by three ELISA systems (Table I), which proved that there was an increase of activity in the eluates and a coincidental decrease of activity in the flow through of the two columns. We were able to quantitatively separate 10% of bispecific monovalent antibodies from falsely recombined molecules, a yield which correlates with the theoretical considerations of Milstein and Cuello (1983). Ninety-five per cent of the bispecific molecules were immunoreactive as revealed by a quantitative binding assay in antigen excess (Table II). Compared to other methods (Doussal *et al.*, 1989; Lenz & Weidle, 1990) this purification method leads to exceptionally high yields. Disadvantageous was the yield of the BHK cells secreting the immunoglobulins: from a cell line which had produced $4-8\,\mu g\,ml^{-1}$ in the beginning, we only harvested about 30 mg immunoglobulin from 10 l supernatant instead of 40–80 mg expected. It has to be accepted that to some extent the cells lost the specificity of antibody production. In another case of antibody production in BHK cells in our laboratory it was revealed that the cells produced much more light chains than heavy chains (Bosslet, unpublished data), a fact which leads to reduced yields of intact molecules. Combined with the fact that two affinity chromatography columns are necessary for purification, these conditions are unfavourable for larger production scales.

Comparison of the humanised bsMAb and the humanised monospecific anti-CEA antibody in SDS-PAGE proved that both molecules resemble each other to a large extent. The most obvious differences appear in the behaviour of the light chains of both antibodies which point to the presence of two different κ-chains in the bsMAb (Figure 2, lanes 5 and 6). This may be due to the fact that one of the light chains still has a whole murine variable region. The integrity of the bispecific MAb was further proven by our immunoreactivity studies indicating that >95% of the bispecific humanised MAb were functionally active. This immunoreactivity is only marginally inferior to the value generated using the humanised monospecific MAb (>96%·.

The anti DTPA-Y could only be evaluated qualitatively. Data from the competitive ELISA system in which the binding of the bsMAb to solid phase attached HSA-benzyl DTPA-Y was completely blocked by a >100 fold excess of free DTPA-Y, argue for the functional integrity and specificity of those bsMAb molecules which bound to the solid phase attached HSA-benzyl DTPA-Y. This type of analysis is less quantitative than the immunoreactivity assay performed for the anti CEA arm, but is nevertheless suited to support the usefulness of the hu bsMAb.

The bispecific MAb generated is intended to be employed in two phase radioimmunotherapy, having dual specificity for carcino-embryonic antigen (CEA) and a radiolabelled chelate (DTPA-$^{90}$Y). Concerning the specific tumour localisation, Bosslet *et al.* (1991) showed that it is possible to obtain a significant tissue penetration of solid human carcinoma xenografts after long time application of the murine anti-CEA MAb BW 431. Since the two phase therapy concept is built up on repetitive long-term and high dose injections of bispecific antibodies, immunisation of patients is most probable. The humanised bsMAb is hoped to overcome the problem of immunogenicity currently seen with murine and chimeric antibodies used in human therapy (Brüggemann *et al.*, 1989).

Considering the yields and the cumbersome purification procedure, attempts have been made to improve the yield of bispecific monovalent MAb using recombinant DNA technology, e.g. the construction of an Ig heavy chain, consisting of $V_H$ of MAB 1 and a $C_H1$ domain, which is linked by a polypeptide spacer to the other heavy chain, consisting of $V_H$ of MAb 2 and a $C_H3$ domain, to form a 'tandem heavy chain'. The association of such a 'tandem heavy chain' with the two corresponding light chains ($V_K$ of MAb 1 and a $C_x$ domain or $V_K$ of MAb 2 and a $C_H3$ domain) should lead predominantly to the formation of the desired bispecific molecule.

The technical assistance of N. Döring, E. Herz, H. Lind and M. Matthäi as well as the secretarial assistance of S. Lehnert are greatly appreciated.

### References

BAGSHAWE, K.D., ROGERS, G.T. & SHARMA, S.K. (1989). Further improvements relating to drug delivery systems. *WO* 89/10140.

BOSSLET, K., STEINSTRAESSER, A., SCHWARZ, A., HARTHUS, H.P., LÜBEN, G., KUHLMANN, L. & SEDLACEK, H.H. (1988). Quantitative considerations supporting the irrelevance of circulating serum CEA for the immunoscintigraphic visualization of CEA expressing carcinomas. *Eur. J. Nucl. Med.*, **14**, 523–528.

BOSSLET, K., KEWELOH, H.C., HERMENTIN, P., MUHRER, K.H., SEDLACEK, H.H. & SCHULZ, G. (1990). Percolation and binding of monoclonal antibody BW 494 to pancreatic carcinoma tissues during high dose immunotherapy and consequences for future therapy modalities. *Br. J. Cancer*, **62**, Suppl. X, 37–39.

BOSSLET, K., STEINSTRAESSER, A., HERMENTIN, P., KUHLMANN, L., BRUYNCK, A., MAGERSTAEDT, M., SEEMANN, G., SCHWARZ, A. & SEDLACEK, H.H. (1991). Generation of bispecific monoclonal antibodies for two phase radioimmunotherapy. *Br. J. Cancer*, **63**, 681–686.

BOULAINNE, G.L., HOZUMI, N. & SHULMAN, M.J. (1984). Production of functional chimeric mouse/human antibody. *Nature*, **312**, 643–646.

BRENNAN, M., DAVISON, P.F. & PAULUS, H. (1985). Preparation of bispecific antibodies by chemical recombination of monoclonal immunoglobulin G1 fragments. *Science*, **229**, 81–83.

BRÜGGEMANN, M., WINTER, G., WALDMANN, H. & NEUBERGER, M.S. (1989). The immunogenicity of chimeric antibodies. *J. Exp. Med.*, **170**, 2153–2157.

DYKES, P.W., BRADWELL, A.R., CHAPMAN, C.E. & VAUGHAN, A.T.M. (1987). Radioimmunotherapy of cancer: clinical studies and limiting factors. *Cancer Treat. Rev.*, **14**, 87–106.

DOUSSAL, J.M., LE, MARTIN, M., GAUTHERAT, E., DELAAGE, M. & BARBET, M. (1989). *In vitro* and *in vivo* targeting of radiolabeled monovalent and divalent haptens with dual specificity monoclonal antibody conjugates: enhanced divalent hapten affinity for cell bound antibody conjugates. *J. Nucl. Med.*, **30**, 1358–1366.

EIJK, H.G. VAN & NOORT, W.L. VAN (1976). Isolation of rat transferrin using CNBr-activated sepharose 4B. *J. Clin. Chem. Clin. Biochem.*, **14**, 475–478.

GOLDSTEIN, G. (1987). Overviews of the development of orthoclone OKT3: monoclonal antibody for therapeutic use in transplantation. *Transplant. Proc.*, **XIX**, 1–6.

GRAHAM, F.L. & EB, A.J. VAN DER (1973). A new technique for the assay of infectivity of human adenovirus 5′ DNA. *Virology*, **52**, 456–467.

GREENMAN, R.L., SCHEIN, R.M.H., MARTIN, M.A., WENZEL, R.P., MACINTYRE, N.R., CHMEL, E.H., KOHLER, R.B., MCCARTHY, M., PLOUFFE, J., RUSSELL, J.A. & THE XOMA SEPSIS STUDY GROUP (1991). A controlled clinical trial of E5 murine monoclonal IgM antibody to endotoxin in the treatment of gram-negative sepsis. *JAMA*, **266**, 1097–1102.

GRITZ, L. & DAVES, J. (1983). Plasmid-encoded hygromycin-B resistance: the sequence of hygromycin-B-phosphotransferase gene and its expression in E. coli and S. cerevisia. *Gene*, **25**, 179–188.

GÜSSOW, D.H. & SEEMANN, G. (1991). Humanization of monoclonal antibodies. *Meth. Immunol.*, **203**, 99–121.

HABER, E., QUERTERMOUS, T., MATSUEDA, G.R., RUNGE, M.S. & BODE, C. (1990). Antibody-targeted thrombolytic agents. *Jap. Circulation J.*, **54**, 345–348.

HALE, G., DYER, M.J.S., CLARK, M.R., PHILLIPS, J.M., MARCUS, R., RIECHMANN, L., WINTER, G. & WALDMANN, H. (1988). Remission induction in non-Hodgkin lymphoma with reshaped human monoclonal antibody CAMPATH-1H. *Lancet*, **ii**, 1394–1399.

HUDZIAK, R.M., LASKI, F.A., RAJBHANDARY, U.L., SHARP, P.A. & CAPECCHI, M.R. (1982). Establishment of mammalian cell lines containing multiple nonsense mutations and functional suppressor tRNA genes. *Cell*, **31**, 137–146.

JONES, P.T., DEAR, P.H., FOOTE, J., NEUBERGER, M.S. & WINTER, G. (1986). Replacing the complementarity-determining regions in a human antibody with those from a mouse. *Nature*, **321**, 522.

JOSEPH, K., HÖFFKEN, H., BOSSLET, K. & SCHORLEMMER, H.U. (1988). In vivo labelling of granulocytes with $^{99m}$Tc anti-NCA monoclonal antibodies for imaging inflammation. *Eur. J. Nucl. Med.*, **14**, 367–373.

KROONENBURGH, M.J.P.C. VAN & PAUWELLS, E.K.J. (1988). Human immunological response to mouse monoclonal antibodies in the treatment or diagnosis of malignant diseases. *Nuclear Med. Commun.*, **9**, 919–921.

KURRLE, R., ENSSLE, K.H. & SEILER, F.R. (1988). Monoclonal antibodies directed to leukocyte differentiation antigens for therapeutic use. *Behring Inst. Mitt.*, **82**, 154–173.

LAEMMLI, U.K. (1970). Cleavage cultures of fused cells secreting antibody of predefined specificity. *Nature*, **227**, 680–685.

LENZ, H. & WEIDLE, U. (1990). Expression of heterobispecific antibodies by genes transfected into producer hybridoma cells. *Gene*, **87**, 213–218.

MILLER, R.A., OSEROFF, A.R., STRATTE, P.T. & LEVY, R. (1983). Monoclonal antibody therapeutic trials in seven patients with T-cell lymphoma. *Blood*, **62**, 988–995.

MILSTEIN, C. & CUELLO, A.C. (1983). Hybrid hybridomas and their use in immunohistochemistry. *Nature*, **305**, 537–540.

MORRISON, S.L., JOHNSON, M.J., HERZENBERG, L.A. & OI, V.T. (1984). Chimeric human antibody molecules: mouse antigen-binding domains with human constant region domains. *Proc. Natl Acad. Sci. USA*, **81**, 6851–6855.

MURRAY, J.L. & UNGER, M.W. (1988). Radioimmunodetection of cancer with monoclonal antibodies: current status, problems, and future directions. *CRC Critical Rev. Oncol./Hematol.*, **8**, 227–256.

ORLANDI, R., GÜSSOW, D.H., JONES, P.T. & WINTER, G. (1989). Cloning immunoglobulin variable domains for expression by the polymerase chain reaction. *Proc. Natl Acad. Sci. USA*, **86**, 3833–3837.

RIECHMANN, L., CLARK, M., WALDMANN, H. & WINTER, G. (1988). Reshaping human antibodies for therapy. *Nature*, **332**, 323–327.

SONGSIVILAI, S., CLISSOLD, P.M. & LACHMANN, P. (1989). A novel strategy for producing chimeric bispecific antibodies by gene transfection. *Biochem. Biophys. Res. Commun.*, **164**, 271–276.

STANLEY, C.J., PARIS, F., PLUMB, A., WEBB, A. & JOHANNSON, A. (1985). Enzyme amplification: a new techniqe for enhancing the speed and sensitivity of enzyme immunoassays. *Int. Comm. Rad. Protect.*, **3**, 44–47.

SUBRAMANI, S., MULLIGAN, R. & BERG, P. (1981). Expression of the mouse dihydrofolate reductase complementary deoxyribonucleic acid in simian virus 40 vectors. *Mol. Cell. Biol.*, **1**, 854–864.

THOMAS, G.D., CHAPPELL, M.J., DYKES, P.W., RAMSDEN, D.B., GODFREY, K., ELLIS, J.R.M. & BRADWELL, A.R. (1989). Effect of dose, molecular size, affinity, and protein binding on tumor uptake of antibody or ligand: a biomathematical model. *Cancer Res.*, **49**, 3290–3296.